# Identification of abdominal aortic aneurysm subtypes based on mechanosensitive genes

**DOI:** 10.1371/journal.pone.0296729

**Published:** 2024-02-09

**Authors:** Chang Sheng, Qin Zeng, Weihua Huang, Mingmei Liao, Pu Yang

**Affiliations:** 1 Department of Vascular Surgery, Xiangya Hospital, Central South University, Changsha, Hunan, China; 2 National Health Commission Key Laboratory of Nanobiological Technology, Xiangya Hospital, Central South University, Changsha, Hunan, China; 3 Department of Clinical Pharmacology, Hunan Key Laboratory of Pharmacogenetics, Xiangya Hospital, Central South University, Changsha, Hunan, China; 4 Institute of Clinical Pharmacology, Hunan Key Laboratory of Pharmacogenetics Xiangya Hospital, Central South University, Changsha, Hunan, China; 5 National Clinical Research Center for Geriatric Disorders, School of Pharmacy, Hunan University of Chinese Medicine, Changsha, Hunan, China; 6 National Clinical Research Center for Geriatric Disorders, Xiangya Hospital, Central South University, Changsha, Hunan, China; Scuola Superiore Sant’Anna, ITALY

## Abstract

**Background:**

Rupture of abdominal aortic aneurysm (rAAA) is a fatal event in the elderly. Elevated blood pressure and weakening of vessel wall strength are major risk factors for this devastating event. This present study examined whether the expression profile of mechanosensitive genes correlates with the phenotype and outcome, thus, serving as a biomarker for AAA development.

**Methods:**

In this study, we identified mechanosensitive genes involved in AAA development using general bioinformatics methods and machine learning with six human datasets publicly available from the GEO database. Differentially expressed mechanosensitive genes (DEMGs) in AAAs were identified by differential expression analysis. Molecular biological functions of genes were explored using functional clustering, Protein–protein interaction (PPI), and weighted gene co-expression network analysis (WGCNA). According to the datasets (GSE98278, GSE205071 and GSE165470), the changes of diameter and aortic wall strength of AAA induced by DEMGs were verified by consensus clustering analysis, machine learning models, and statistical analysis. In addition, a model for identifying AAA subtypes was built using machine learning methods.

**Results:**

38 DEMGs clustered in pathways regulating ‘Smooth muscle cell biology’ and ‘Cell or Tissue connectivity’. By analyzing the GSE205071 and GSE165470 datasets, DEMGs were found to respond to differences in aneurysm diameter and vessel wall strength. Thus, in the merged datasets, we formally created subgroups of AAAs and found differences in immune characteristics between the subgroups. Finally, a model that accurately predicts the AAA subtype that is more likely to rupture was successfully developed.

**Conclusion:**

We identified 38 DEMGs that may be involved in AAA. This gene cluster is involved in regulating the maximum vessel diameter, degree of immunoinflammatory infiltration, and strength of the local vessel wall in AAA. The prognostic model we developed can accurately identify the AAA subtypes that tend to rupture.

## 1. Introduction

Abdominal aortic aneurysm (AAA) is defined as the progressive segmental dilation of the abdominal aorta [1]. Hypertension, male sex, advanced age (>65 years), smoking, coronary artery disease, family history of AAA, and peripheral arterial disease are risk factors for AAA [1]. Small aneurysms are at a low risk of rupture, a priority recommendation for regular follow-up, but there are currently no drugs that are effective in limiting aneurysm enlargement and aneurysms >5.4 cm in diameter require elective repair [2–4]. A large prospective cohort study indicates that, for non-ruptured AAA patients, there has been little change in perioperative mortality rates in recent years for two surgical treatment modalities: open surgical repair (OSR) and endovascular aneurysm repair (EVAR), with rates of 4.6% and 1.3%, respectively [5].

Current evidence suggests that AAA results from an altered hemodynamic microenvironment and immune response (local and systemic) [6]. Mechanical and frictional forces play an important role in the development of AAA [7]. Complex blood flow patterns and wall shear stress (WSS) in the vasculature are thought to be associated with vascular dysfunction, which may alter endothelial cell function by regulating the expression of cellular genes in different regions of the arterial wall [8, 9]. Endothelial dysfunction is an early pathological event in AAA formation that promotes oxidative stress and inflammation of the degenerating arterial wall [10]. WSS regulates vascular endothelial function by acting on many shear-sensitive genes such as JNK, HIF-1α, NF-κB, KLF2/4, and NRF2 [11, 12]. Increased expression of HIF-1α in human AAA and HIF-1α inhibitors can restrict experimental AAA progression [13]. In addition, WSS in the bloodstream can cause significant effects on smooth muscle physiological functions through heparan sulfate proteoglycans, ERK1/2, and Tissue factor pathway inhibitor-2 [14, 15]. Biomechanical environments such as blood flow shear, blood pressure and extracellular interstitial stiffness can also regulate monocyte/macrophage adhesion, migration and retention [16]. In AAA, the genes that respond to altered hemodynamics have not been well identified and explained. We speculate that the regulation of mechanosensitive genes may play an essential role in accelerating AAA.

Molecular and histopathological evaluation of abdominal aortic tissue samples provides a comprehensive approach for identifying mutually coordinated gene expression in the biological pathways involved in AAA. The purpose of this study was to identify mechanosensitive genes and important mediating pathways in AAA pathological tissues by combining microarray data with bioinformatics techniques. Furthermore, AAA subgroups were identified based on the expression patterns of mechanosensitive genes, and differences between these subgroups were characterized. Finally, a prediction model was constructed by combining machine learning methods.

## 2. Materials and methods

### 2.1 Data collection and processing

**Fig 1:**
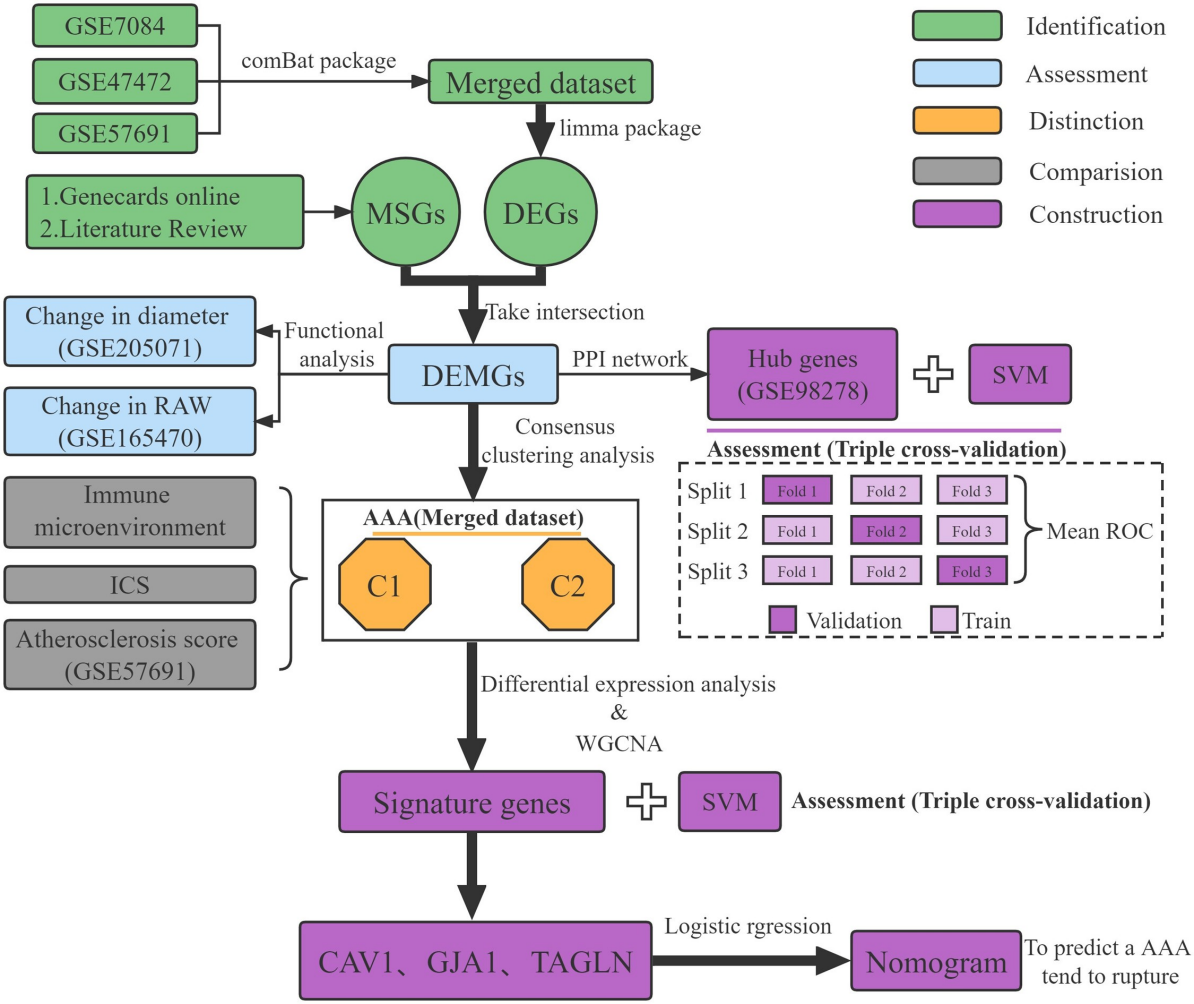
Flowchart of this study. DEMGs, differentially expressed mechanosensitive genes; RAW, Regional Aortic Weakness; SVM, support vector machine; ICS, inflammatory composite score.

A workflow of this study is shown in Fig 1. The GEO database (http://www.ncbi.nlm.nih.gov/geo/) was searched for gene expression in human AAAs. Six datasets were identified and analyzed in the present study. These include GSE7084, GSE47472, GSE57691, GSE205071, GSE165470, and GSE98278 (S1 Table in S1 File). Datasets (GSE7084, GSE47472, and GSE57691) with insufficient demographic information were combine into larger dataset using the comBat algorithm, an analysis method based on classical Bayesian [17]. All cases enrolled in GSE47472 study were added to the control group because the study only included non-aneurysmal aortic tissues. Principal component analysis (PCA) was used to access data distribution across the three datasets. The GSE205071, GSE165470, and GSE98278 studies contained more clinical information such as sex, age, and aneurysmal diameter. Hence, this data was reserved to authenticate the functions of differentially expressed mechanosensitive genes (DEMGs). Mechanosensitive genes (MSGs) were downloaded from GeneCards (https://www.genecards.org/) using the search strategy "[all] (mechanosensitive) OR [all] (vascular AND shear AND stress)”. The selected genes were sorted by their relevance score, and genes ranked top 25% were deemed as mechanosensitive genes. In addition, literatures were searched for validated mechanosensitive genes that are missing from the list [11, 12, 14, 15]. A total of 215 genes were selected for further analyses (S2 Table in S1 File).

Genes meeting an adjusted P-value of less than 0.05 and |log2 (fold change (FC))| > 1.5 were defined as differentially expressed genes (DEGs) [18]. DEMGs were identified by intersecting DEGs with MSGs (https://bioinformatics.psb.ugent.be/webtools/Venn/). The DEMGs were then subjected to functional enrichment and protein-protein interaction (PPI) analyses. PPI analysis was set with an interaction score of 0.4 [19, 20] and exclusion of hidden disconnected nodes. The interaction data were imported into Cytoscape to find hub genes based on Degree, Maximum Neighborhood component (MNC), Closeness, and Maximal Clique Centrality (MCC) algorithms. The iRegulon plugin was used to screen the key transcription factor (TF) with the cutoff values recommended by others [21].

### 2.2 Identification and Exploration of mechanosensitive AAA subgroups

Initially, the power of the DEMGs was evaluated using consensus clustering analysis (an unsupervised classification method) in combination with demographic data. In the GSE205071 dataset, each sample contained information on sex, age, and maximum AAA diameter. Since the majority of AAA cases were male in this study, only data for male patients were analyzed. The GSE165470 study quantified the Regional Aortic Weakness (RAW) for all AAAs as an indicator for assessment. Subsequent consensus clustering analysis of AAA samples in the merged dataset was based on DEMGs. Genes meeting an adjusted P-value of less than 0.05 and |log2 (fold change (FC))| > 1.5 were defined as subtype differentially expressed genes (sDEGs).

Immune-mediated infiltration plays a significant role in the pathogenesis of AAA [22]. The immune cell infiltration fraction for AAA samples in the merged dataset was calculated using the ‘estimate’ package [23]. In addition, immune microenvironment (IME) profiling, including HLA family genes and immune cells [24, 25], was explored between the AAA subgroups (merged dataset).

Separate weighted gene co-expression network analysis (WGCNA) was performed using AAA samples (all genes) from the merged dataset, and genes with the highest 50% variance were filtered. The Pearson correlation matrix of the sample was then calculated. A weighted adjacency matrix was created and subsequently converted into a Topological Overlap Metric (TOM) matrix [26]. Mean chain hierarchical clustering was used to construct a clustering tree diagram of the TOM matrix and the threshold of the merged similarity module was set to 0.25. The common part of the sDEGs, the highest correlated modular genes, and mechanosensitive genes was taken as “signature genes” [26].

Subsequently, a ‘linear’ kernel support vector machine (SVM) was built based on the “signature genes”. Correlative coefficients were used to calculate the mechanical sensitivity score as follows: ΣI Coefficientsi×Expression level of genei [26]. Than, a clinical diagnostic nomogram was created and a calibration curve was used to verify the validity of the diagnostic model. In the nomogram, each gene is given a weight for its ability to distinguish AAA subtypes, allowing the correlation of each gene expression with the score to be determined [27].

Chemical molecular structures of drugs were obtained from PubChem (https://pubchem.ncbi.nlm.nih.gov/). Targets for Nifedipine and Amlodipine were obtained from the SwissTargetPrediction (http://swisstargetprediction.ch/), Targetnet (http://targetnet.scbdd.com/), and GeneCards websites.

### 2.3 Bioinformatics and statistical analysis

The relevant libraries, packages, and algorithms used in this study are presented in S3 Table in S1 File. Briefly, the ‘limma’ R algorithm was used to identify DEGs. Gene Ontology (http://geneontology.org/) and pathway enrichment analysis were performed to explore potential biological functions and pathways. Pearson’s and Spearman’s correlation tests were used to evaluate associations. The chi-square test and t-test or Mann–Whitney U-test were used to compare categorical and continuous data in different groups. P or adjusted P (Benjamini and Hochberg method) <0.05 was considered statistically significant.

## 3. Results

### 3.1 Identification of DEMGs

**Fig 2:**
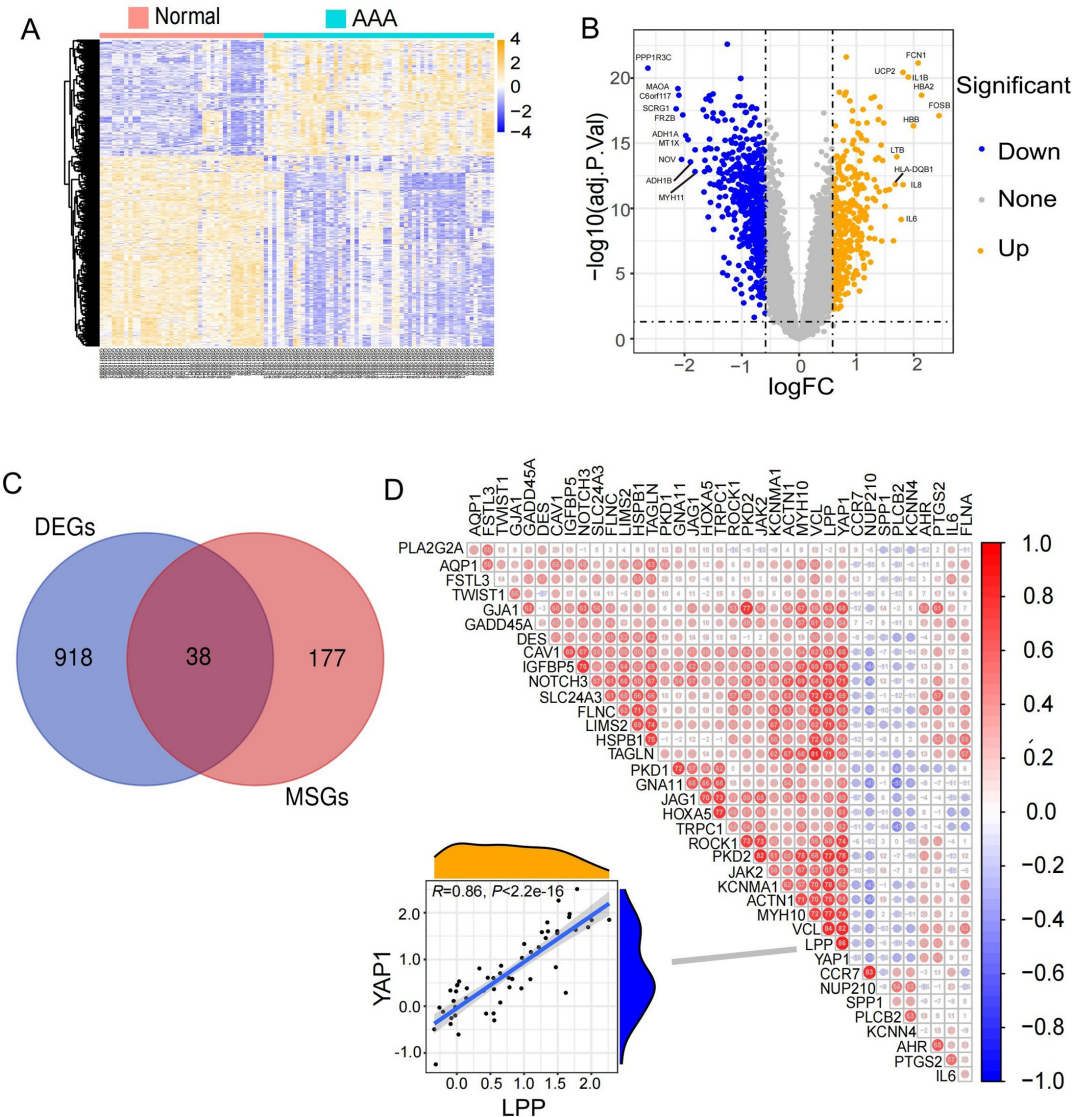
Analysis for DEGs and correlation analysis between DEMGs. (A) Expression level heatmap and hierarchical clustering of DEGs in the merged dataset. (B) Volcano plot of DEGs in the merged dataset. (C) The Venn diagram shows the common parts of DEGs and mechanosensitive genes as DEMGs. (D) Heatmap of correlation between DEMGs and scatter plot of highest correlation group. The color shades and circle sizes in the heatmap represent the magnitude of the absolute value of the correlation coefficient. Each point in the scatter plot represents a AAA sample, and the wave crest graph shows the distribution of gene expression data.

PCA of the merged dataset showed a fairly homogenous data distribution for individual datasets as well as for the AAA cases and control samples (**S1 Fig** in S1 File). A total of 956 DEGs (361 upregulated and 595 downregulated genes) were identified for AAAs (Fig 2A and 2B). Evidently, among the DEGs, more genes are down- than up-regulated in AAA cases. A total of 38 DEMGs were found among the DEGs, of which 8 were upregulated and 30 were downregulated (Fig 2C and **S4 Table** in S1 File). This findings is consistent with previous reports by other groups, showing differential expression *ACTN1*, *FLNA*, *LPP*, *HSPB1*, *IL6*, *PLCB2*, *PTGS2*, and *SPP1* [28, 29], as well as *SPP1* between AAA patients and control aortas [29]. We calculated the correlation between the DEMGs, and most of the genes were highly associated with positive correlation in their expression (Fig 2D), suggesting that genes functions in a modular fashion, carrying out specific activities. Therefore, in the next step, we performed functional enrichment analysis of the DMEGs to identify key biological processes.

### 3.2 The efficacy of DEMGs to identify predetermined AAA subgroups

**Fig 3:**
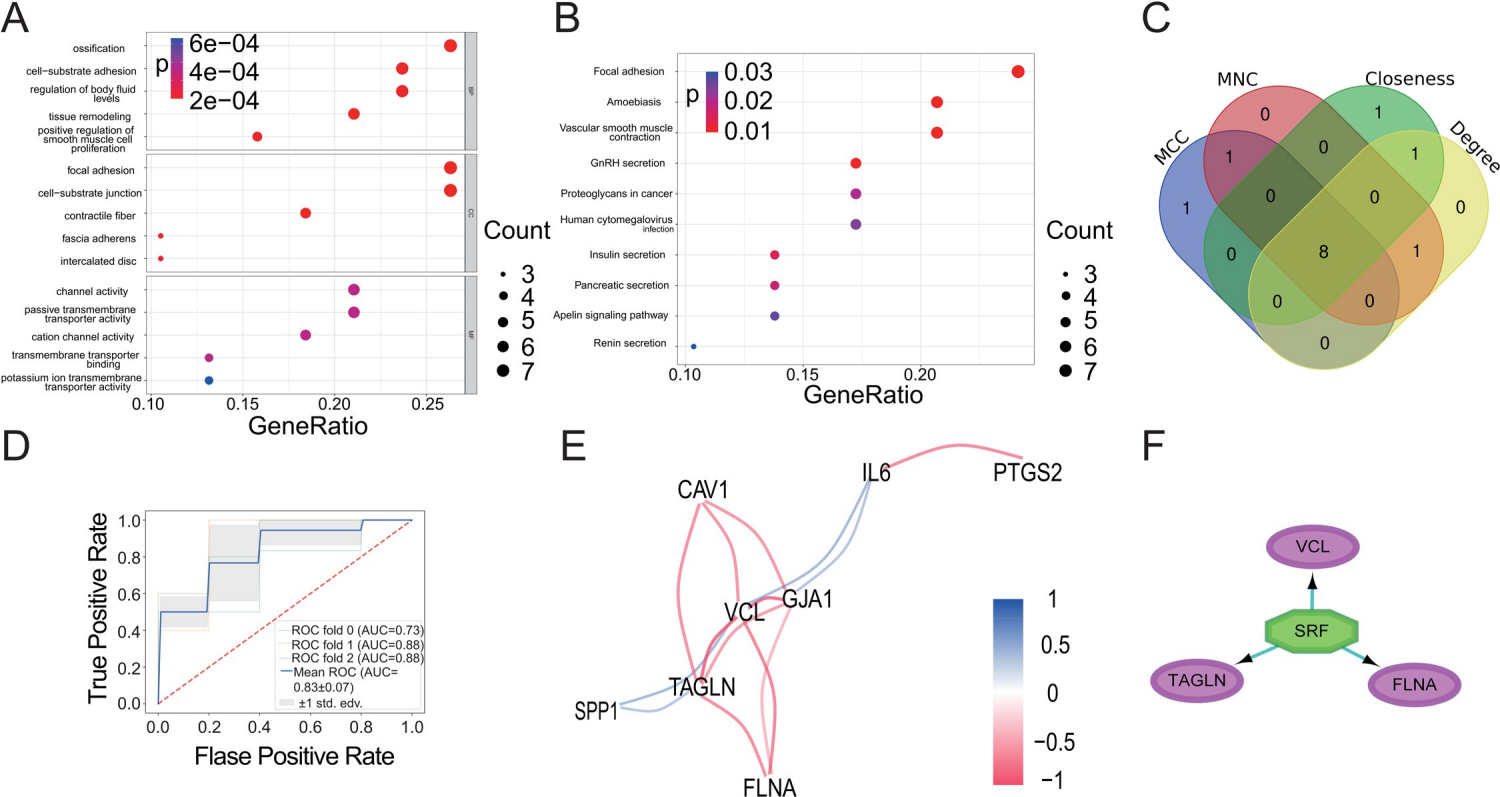
Enrichment analysis of DEMGs and validation and interaction analysis of hub genes. (A, B) GO and KEGG enrichment analysis of DEMGs. € The Venn diagram shows the overlap of results generated by four different algorithms to obtain hub genes. (D) ROC curves for Triple cross-validation show the strong performance of the ‘rbf’ SVM model in GSE98278 (large- and intermediate-sized AAA). (E) Representative gene relationship network diagram of hub genes. Connections with a correlation of less than 0.3 are not shown. The shade of color indicates the absolute value of the correlation coefficient. (F) The transcription factor SRF regulates three hub genes.

Gene Ontology analysis revealed that DEMGs were highly enriched in functions related to ‘Phenotypic transformation of vascular smooth muscle cells,’ ‘Cell or Tissue connectivity’, and ‘Channel activity’ (Fig 3A and 3B). While this finding is not surprising, it underscores the importance of the cellular contractile apparatus in mechanosensing and AAA development, as seen in the formation of thoracic aortic aneurysms and dissections [30]. We screened possible targets of the L-type calcium channel blockers Nifedipine and Amlodipine at DEMGs. A total of 365 Nifedipine and 190 Amlodipine targets were obtained from the database as specified in the “Methods”. Eight nifedipine targets and three Amlodipine targets were found as DEMGs, among which *JAK2* and *AHR* were down- and up-regulated, respectively, in AAAs (Table 1).

**Table 1:** Drug target genes among DEMGs (Common targets in bold).

Drugs	Genes
Nifedipine	**JAK2 AHR** KCNMA1 GJA1 SPP1 PTGS2 PLA2G2A TRPC1
Amlodipine	**JAK2 AHR** IL6

The PPI networking of the 38 DEMGs was evaluated using the STRING database and the Cytoscape software. With a cut-off value of 0.4, 34 of 38 DEMGs were predicted to interact each other (**S2 Fig** in S1 File), indicating that these genes work together in response to aneurysmal degeneration. Within this network, hub genes were identified using four topological complementary analysis methods as reported by others [31, 32]. A total of eight hub genes were identified (Fig 3C and **S5 Table** in S1 File).

To evaluate the clinical relevance of the hub genes, we tested whether their expression levels correlate with predetermined, clinically meaningful AAA subgroups. The GSE98278 study was chosen for the analysis because it categorized AAAs as “large-sized” and “intermediate-sized” subgroups during patient enrollment [33]. After testing multiple SVM models with different computational kernels, the ‘rbf’ SVM presented the best performance. The hub genes enabled the machine learning model to achieve dichotomous classification of large vs. intermediate-sized AAAs. When the true positive rate reaches 80%, the false positive rate remains to be quite low (around 20%) (Fig 3D).

When the correlation coefficient is less than 0.3, the probability of correlation is considered small [34]. With this cutoff value, correlation analysis for the hub genes showed that *TAGLN*, *VCL*, and *GAJ1* were positioned as the center nodes (Fig 3E). We further searched for upstream regulators for the hub genes using the iRegulon plugin. The results showed that *SRF*, a master transcriptional for smooth muscle cell contractile genes [35], serves as the critical regulator for the hub genes *TAGLN*, *VCL*, and *FLNA* (Fig 3F).

### 3.3 The efficacy of DEMGs to predict clinically meaningful AAA subgroups

With the available clinical data from the database, we evaluated DEMGs for their efficacy to discriminate clinically meaningful AAA subgroups. Consensus clustering analysis was performed for the GSE205071 and GSE165470 datasets to assign AAAs to subgroups based on their DEMGs expression profile. For the GSE205071 dataset, the case GSM6204818 was removed due to the serious presence of data outliers. Consensus clustering analysis divided AAA cases enrolled to the GSE205071 study to two subgroups (C1 and C2) (**S3A-S3C Fig** in S1 File **and S3E and S3F Fig** in S1 File**).** Intriguingly, further analysis of the clinical data revealed a significant difference in standard diameter of the AAAs between the two subgroups (**S3D Fig** in S1 File). Similarly, consensus clustering analysis assigned AAA cases in the GSE165470 dataset to two subgroups (**S4A-S4F Fig** in S1 File **)**. Further analysis of the clinical data found that AAAs in the subgroup C1 had a significantly larger reginal aortic weakening index (RAW) than that of the C2 (**S4D Fig** in S1 File). These results demonstrate that levels of DEMGs are associated with meaningful clinical features of AAAs.

Next, we looked into the expression of hub genes in the C1 and C2 subgroups for both the GSE205071 and the GSE165470 datasets. Subgroup-dependent differential expression of the hub genes was found for the GSE205071 (**S5A Fig** in S1 File) and the GSE165470 (**S5B Fig** in S1 File) datasets. Expression of the hub genes in AAA cases and control samples assigned to the merged dataset was also analyzed (**S5C Fi**g in S1 File). Note the similarity of expression profile of the hub genes between AAA subgroups and between AAAs and non-aneurysm controls. It appears that the hub genes have similar efficacy to identify AAAs with a greater risk of clinical complications and to discriminate AAA cases from non-aneurysm controls, as compared to entire set of the DEMGs can separate AAA into two groups with good and poor characteristics. Therefore, the next step was to perform a formal subgroup analysis in the merged dataset.

### 3.4 Subgroup analysis of AAA in the merged dataset

**Fig 4:**
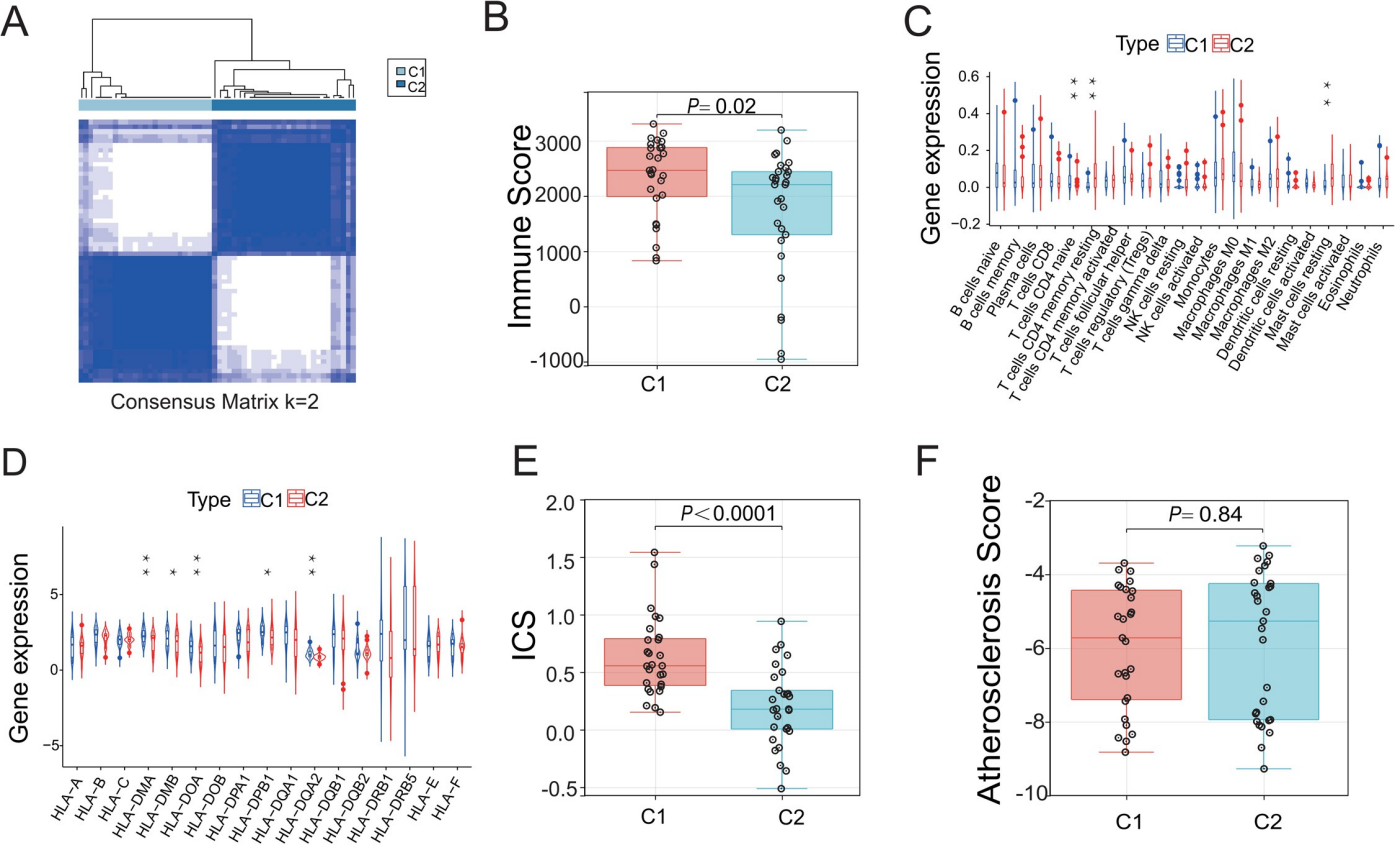
Establishment of AAA subgroups and comparison of differences between the subgroups for the merged dataset. (A) Heatmap of the co-occurrence ratio matrix of AAA samples (k = 2). (B) Significant differences in Immune scores between AAA subgroups. (C) The violin plot shows the difference in the abundance of immune infiltrating cells between subgroups. (D) The violin plot shows the difference in HLA family gene expression between subgroups. (E) ICS was significantly different in the two subgroups. (F) The atherosclerosis score was similar in both subgroups. *P < 0.05, **P < 0.01, ***P < 0.001, Blank: no significance.

The sufficient efficacy of DEMGs in predicating clinically meaningful AAA subgroups in the two separate, small-scale studies promoted us to further evaluate their performance with the merged dataset. Consensus clustering analysis with a K of 2, 3, 4, and 5 showed that AAA samples in the merged dataset may be best grouped to two subgroups, labeled as C1 and C2 (Fig 4A and **S6A-S6C Fig** in S1 File). Functional analysis revealed a significantly higher score in immune cell response for the C1 subgroup compared with the C2 subgroup, indicating more intensive inflammation exists in the C1 than in the C2 subgroups (Fig 4B and **S6 Table** in S1 File). Additionally, expression of genes related to naive CD4 T cells, memory-resting CD4 T cells, and resting mast cells is significantly different in the C1 than in the C2 subgroups (Fig 4C). *HLA-DMA*, *HLA-DMB*, *HLA-DOA*, *HLA-DPB1*, and *HLA-DQA2* were expressed at a significantly higher level in the C1 than in the C2 subgroups (Fig 4D). All these genes are essential components of the HLA class II complex that functions to present antigens to CD4+ T cells during adaptive immune responses. It appears that adaptive immune responses counted for the differential immune score between the C1 and the C2 subgroups. This finding is consistent with previous reports that the mediators produced by CD4+ T cells are involved in the pathogenesis of aneurysmal lesions [36].

To further characterize the C1 and the C2 subgroups, we compared the degree of inflammation and atherosclerosis between these subgroups. The degree of the inflammation is indexed with the vascular inflammatory composite score (ICS). It is documented that ICSs correlate well with the histological and morphological features of AAAs [37]. Using the same method, we calculated the ICS for each sample and found that the C1 subgroup had a higher ICS than the C2 subgroup (Fig 4E and **S7 Table** in S1 File).

The GSE57691 dataset contains non-aneurysmal aortic samples collected from patient with lower extremity arterial occlusive disease (AOD) due to arterial atherosclerosis. Taking advantage of this data, we evaluated atherosclerosis score for these non-aneurysmal aortas and AAAs. The method for assessing atherosclerosis score was developed based on a gene expression matrix of samples, with technical details shown in the **S8 Table** in S1 File **and S7 Fig** in S1 File. When applied to AAA samples, the analysis yielded similar atherosclerosis scores for the C1 and the C2 subgroups (Fig 4F), indicating that atherosclerosis is not a significant contributor to the differential molecular signature presented by the two subgroups. The relationship between AAA and atherosclerosis is unclear [38], and this result does not support atherosclerosis as an influential factor in AAA.

### 3.5 Construction of a mechano-sensitivity scoring system for subgrouping of AAAs

**Fig 5:**
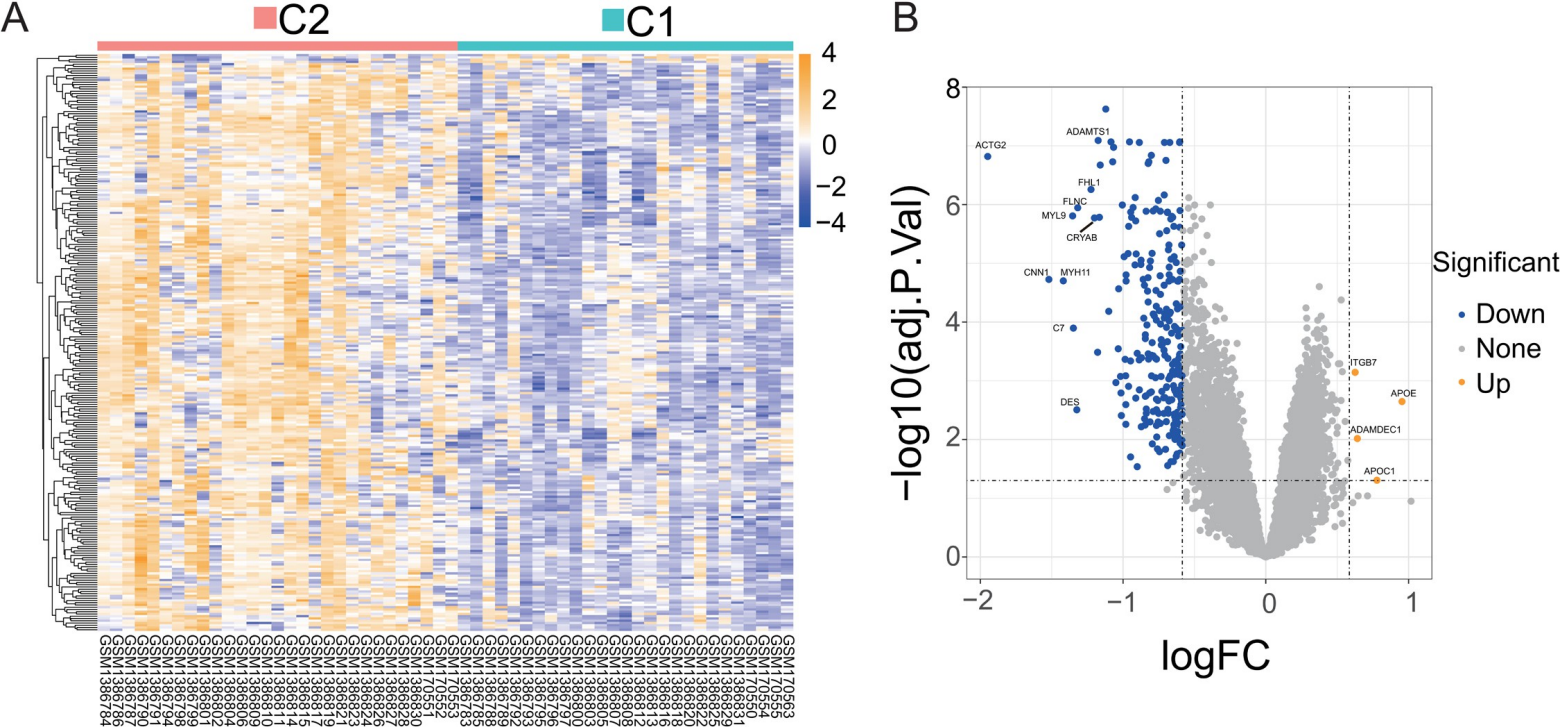
Identification of DEGs between AAA subgroups. (A) Expression level heatmap and hierarchical clustering of DEGs among subgroups. (B) Volcano plot of DEGs among subgroups.

**Fig 6:**
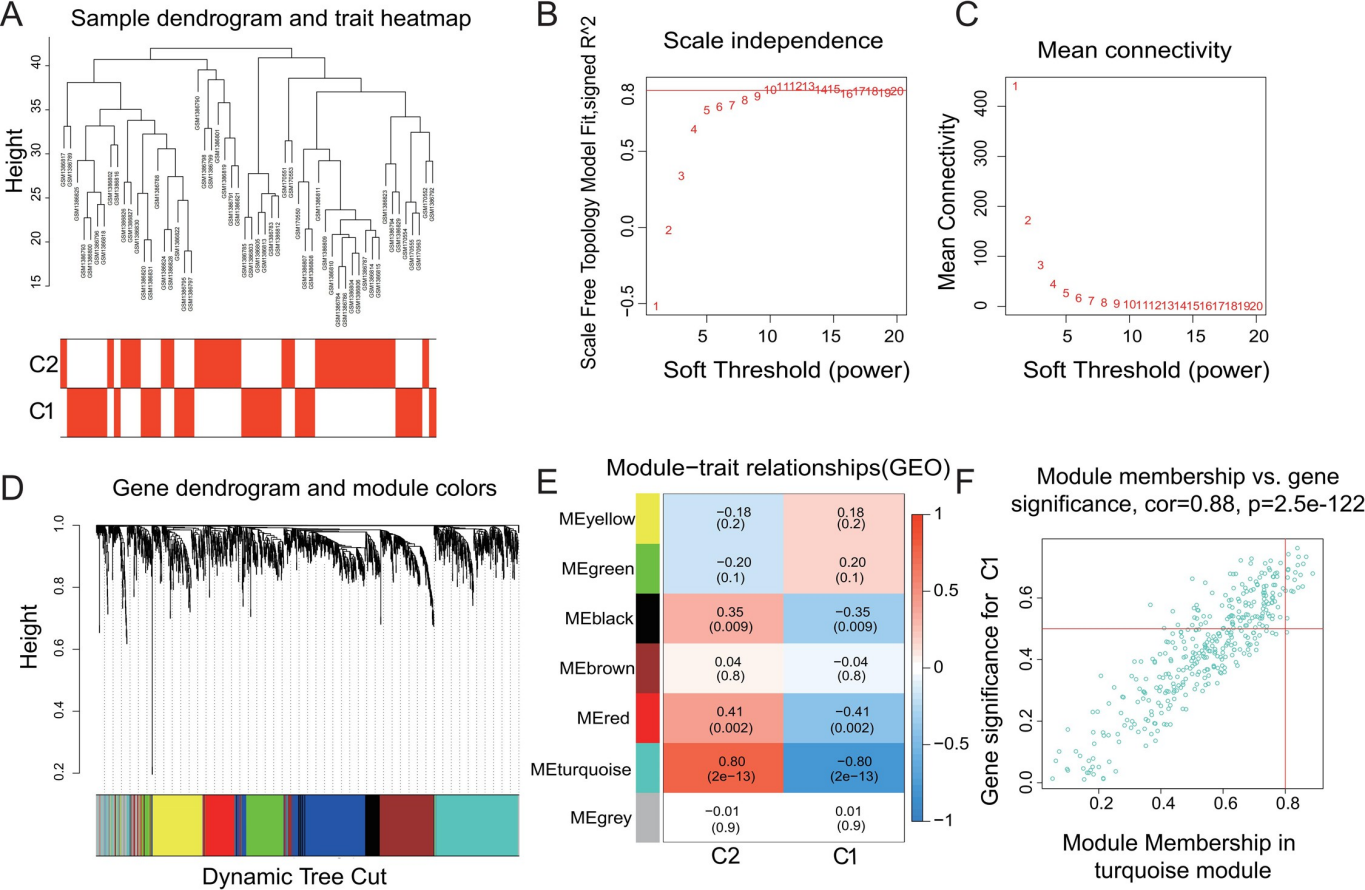
WGCNA on AAA conditions. (A) Based on the full gene expression of the AAA case, average linkage hierarchical clustering analysis revealed no outliers. (B) and (C) Soft-thresholding power analysis was used to obtain the scale-free fit index of network topology, and the optimum soft threshold is 10. (D) Hierarchical cluster analysis was conducted to detect co-expression clusters with corresponding color assignments. Each color represents a module in the constructed gene co-expression network. (E) Calculated correlation coefficients between the modules and subgroups. The magnitude of the correlation is indicated by the shade of the color. (F) Significant correlation existed in the module membership (MM) and gene significance (GS) of the turquoise module.

As presented above, consensus clustering analysis separated the AAAs in the merged dataset to two subgroups, differing primarily in the degree of inflammation including adaptive immune responses. Since that analysis takes the entire gene expression profile into account, we wondered whether DEMGs are capable of predicting C1 vs. C2 AAAs. Differential gene expression analysis identified a total of 256 sDEGs were identified, of which 4 were upregulated and 252 were downregulated in C1 (Fig 5A and 5B). Additionally, the full gene expression profile of the AAA samples in merged dataset was analyzed with the WGCNA algorithm to identify co-expression networks and gene modules that are most significantly associated with the C1 and C2 membership among the AAAs. The average linkage hierarchical clustering analysis showed that all samples fell into one of the clusters, with outliers unidentified (Fig 6A). Power-law distributions are the best mathematical way to describe gene family relationships [39]. We evaluated the scale-free topology model fit at various soft thresholding power, and found that, when the scale-free fit index is at 0.8, the minimum soft thresholding power for constructing a scale-free network is 10 (Fig 6B). At this soft threshold, the networks achieve a stable mean connectivity (Fig 6C). With soft threshold of 10, WGCNA analysis identified seven gene clusters (dendrogram, Fig 6D) and the corresponding models (color coded boxes, Fig 6D). The number of genes assigned to each model is as follows: Black module (419 genes), turquoise module (374 genes), brown module (285 genes), yellow module (207 genes), green module (197 genes), red module (128 genes), and grey module (103 genes). Sample trait analysis (i.e C1 vs. C2) was performed using the method described by others [40]. Among these modules, the turquoise module correlated with C1 (R = -0.80) and C2 (R = 0.80) subgroups to the most degree (Fig 6E). In this model, the module membership (MM) correlated strongly with gene significance (GS, cor = 0.88, Fig 6F). Genes with high MM and GS are considered to be at the pivotal position of the module or hub genes [41]. In other words, genes plotted on the upper-right quadrant (Fig 6F) may be highly associated with the immune microenvironment and ICS in AAAs.

To further evaluate the importance of the genes clustering in the turquoise module, we examined overlapping of those genes with sDEGs identified between the C1 and the C2 subgroups and identified DEMGs among the overlapped genes.

**Fig 7:**
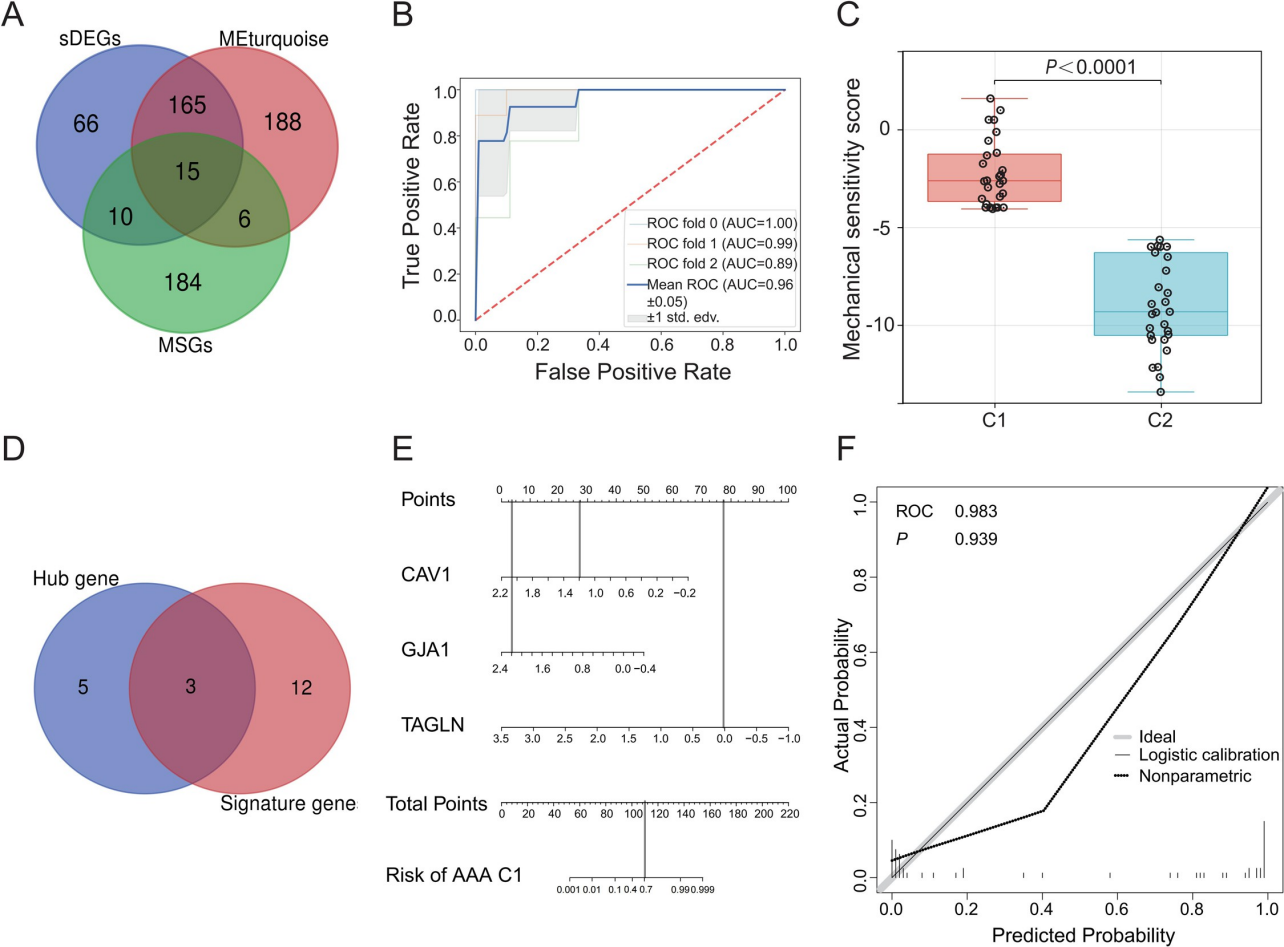
Construction of Mechanical sensitivity score and nomogram. (A) Venn diagram showing overlapping of three gene sets to obtain the signature genes. (B) ROC curves for Triple cross-validation show the strong performance of linear SVM (AAA samples in the merged dataset). (C) C1 had a higher mechanical sensitivity score than C2. (D) Use of hub gene shared with signature gene for the next step of analysis. (E) Construction of clinical diagnostic nomogram based on expression of *CAV1*, *GJA1*, and *TAGLN*. (F) Calibration curve showed the validity of the nomogram (*P* > 0.05).

As shown in the Fig 7A and **S9 Table** in S1 File, there were 15 genes in common among sDEGs, modular genes, and mechanosensitive genes, and they are named as “signature genes”. After testing models with different computational kernels, a “linear” SVM based on the 15 signature genes was found to have the best performance in predicting the C1 vs the C2 subgroups (Fig 7B). This finding suggests that these signature genes are intrinsically important for determining the differences between the two AAA subgroups.

Since all “signature genes” are also mechanosensitive genes, we calculated the mechanical sensitivity score using the function as follows: Mechanical sensitivity score = (-0.1638719 × ACTN1) + (-0.71477884 ×CAV1) + (-0.50420855 × DES) + (-0.41167134 × EDN1) +(-1.09902697 × FLNC) + (-0.37698051 × GADD45A) + (-1.60272468 ×GJA1) + (-0.23867712 ×HSPB1) + (-0.63881431 × IGFBP5) + (0.59983533 × LIMS2) +(0.21890766 × LPP) + (-0.12941644 × MYH10) + (0.00968639 ×SLC24A3) + (-0.45400503 × TAGLN) + (-0.93871657 ×YAP1), where numbers indicate weight assigned to individual genes while gene symbol represent the expression level of these genes. We calculated mechanical sensitivity scores for each sample (**S10 Table** in S1 File) and found that mechanical sensitivity scores are significantly different between the C1 and the C2 subgroups (Fig 7C).

Because the “hub genes” identified with the study GSE98278 were able to assign AAAs to predetermined subgroups with a satisfactory accuracy (Fig 3D) and the “signature genes” predicated AAA subgroups differing in the inflammatory state with a high sensitivity and specificity (Fig 7B), we wondered whether there are genes in common between the two lists and what is their efficacy to predict clinically meaningful trait of AAAs. Three genes were identified by crossing the signature genes with the hub genes (Fig 7D). Using these genes, we constructed a nomogram to assess the risk of being the C1 subgroup (Fig 7E). For example, the expression level of 1.2, 2.2, and 0.0 for *CAV1*, *GJA1*, *and TAGLN*, respectively. The corresponding scores are 27.5, 3.75, and 77.5, which yields the total point of 108.75 for this case with the probability of 70% to be a C1 AAA (Fig 7E). The sample is divided into several groups according to the predicted probability of nomogram. Taking the average predicted probability of each group as the abscissa, and actual probability of each group as ordinate, the calibration curve confirmed the validity of the nomogram (*P* > 0.05, Fig 7F). The false negative rate may be elevated in the application of nomogram.

## 4. Discussion

In this study, we identified mechanosensitive genes associated with the onset and progression of AAA. In combination with the demographic data of the sample and other characteristics reflected by the gene set, we performed a robust characterization of the established groups to explain the role of DEMGs. The performance of prediction model in this study is comparable to previous [42–44]. Data is the cornerstone of the study, and the combination of disease information for the rational grouping of subtypes and further validation increases the reliability and validity of the results. Due to the paucity of data on non-oncological diseases, many bioinformatics analyses stop at the identification of subgroups without further characterization. We have made improvements to these two areas.

We report that C1 has higher immune cell scores, ICS, expression of HLA genes, and mechanical sensitivity scores and lower cellular activity. A higher degree of immune cell infiltration and inflammatory response was observed in ruptured AAA than in stable AAA [45]. Histochemical and immunohistochemical analyses have shown that rupture of AAA occurs because of unstable atherom, hypocellularity, and loss of contractile characteristics of smooth muscle cells in the intima and media [46]. The structure and strength of the aortic wall, mechanical characteristics of the aorta, and cellular and proteolytic components of the AAA wall can directly contribute to AAA rupture [47]. Therefore, it is presumed that individuals with higher mechanical sensitivity scores are more prone to the rupture state.

Function enrichment analysis of DEMGs showed that they are associated with smooth muscle cells, cell or tissue connectivity, and channel activity. The important roles of vascular smooth muscle cells (VSMC) in AAA are associated with apoptosis, phenotype transformation, extracellular matrix regeneration and degradation, proliferation, and contraction [48–51]. Studies have suggested that some DEMGs may play a role in AAA. *TAGLN* is an actin-binding protein that regulates VSMC contraction and acts as a marker of the differentiation phenotype [52], post-deficiency promotes apoptosis in VSMC through macrophage activation [53]. *TAGLN* may serve as a promising target for mediating the development of AAA. *CAV1*-enriched caveolae in VSMC mediate *ADAM17* (deintegrin and metalloproteinase 17)-dependent transactivation of *EGFR* (epidermal growth factor receptor), which is associated with AngII-induced vascular remodeling [54]. *SPP1*, also called osteopntin (OPN), is an inflammatory extracellular matrix protein that is secreted by membranous VSMCs in AAA patients, which is elevated in circulating plasma and aortic walls and is involved in the formation and growth of AAA [55]. Cigarette smoke extracts are potent stimulators of OPN secretion by VSMC [55]. In addition, *AQP1* is expressed in human atherosclerotic angiopathy, and the lack of *AQP1* enhances the formation of atherosclerosis in mice [56]. Other unmentioned DEMGs may also provide novel directions for future research. SRF has also been identified as an important transcription factor in the pathogenesis of AAA [57]. Mechanistically, the binding of SRF to its coactivator mediates the stabilization of the VSMC contractile phenotype by BAF60c [58].

AAA is a disease with a marked inflammatory stress response [59–62], and DEMGs involve several inflammatory modulation modalities. The serum EPO concentration in AAA patients is higher than that in healthy patients. EPO can induce endothelial cell proliferation, migration, and angiogenesis through the JAK2/STAT5 signaling pathway, thus inducing the occurrence of experimental AAA, and blockade of JAK2/STAT3 suppresses the growth of AAA [63–65]. *GJA1* is associated with shear stress and inflammation [66, 67]. Currently, it has been more extensively studied in thoracic aortic aneurysm and dissection (TAAD) [68, 69], and the mechanistic role in AAA remains unclear. *NOTCH3* was identified as a DEMG. Smooth muscle cell-specific *NOTCH1*, but not *NOTCH3*, haploinsufficiency regulating *CTGF* expression could limit AAA progression [70]. More research is needed to confirm the role of *NOTCH3* in AAA. In our study, *CCR7* expression was upregulated in AAA patients. *CCR7* has been highlighted as a target for immunotherapy, and its abnormal expression in the dendritic cell leads to dendritic cell migration disorder [71]. As inflammation is considered a central factor in the development of AAA [72], attention should be paid in order to devise the optimal interventions for AAA. After AAA repair, the hormonal and metabolic stress-related inflammatory cascade is clinically referred to as “post-implantation syndrome.” Circulating IL-6 is a marker of the inflammatory response after EVAR and may be a useful predictor of the occurrence of “post-implantation syndrome” [73].

Genome-wide association studies (GWAS) have been used to identify genomic variants associated with the risk of disease or specific traits [74]. Based on these large-scale studies, *JAK2* and *YAP1* have genome-wide significance for AAA [75, 76]. In addition, *TWIST1* expression affects the vascular smooth muscle cell phenotype, proliferation, and calcification [76], which may also serve as a potential mechanism to support the role of *TWIST1* in AAA. The genes in the DEMGs also play other roles; for example, *IL6*, *CAV1*, *PTGS2*, and *HSPB1* are thought to be key ferroptosis-related genes involved in AAA formation and rupture [77]. This in some way to explains its importance in AAA.

There are some limitations in our study. Firstly, as the controls in the database were mostly donors, this study is at risk of uncertainty regarding many contextual factors in the search for DEMGs. For example, the age and gender of many samples is unclear. Secondly, the most reliable predictor of AAA rupture is the maximum AAA diameter [47]. In our study on the GSE205071 dataset, no differences were found in the unstandardized maximum diameter between groups. This result may be limited by the sample size or unevenness in the duration of AAA, standardized maximum diameters are required to distinguish between nuanced risk subgroups in the AAA population. Thirdly, although the DEMGs also correlate well with each other, the patterns of mutual regulation have not been well analyzed. In addition, some additional minor shortcomings are as follows. DEMGs in AAA may be involved in regulating changes in AAA wall RAW. However, the sample size limits the strength of this evidence. It has been demonstrated that using MCC to predict essential proteins in yeast has the best accuracy [32]. However, the strengths and weaknesses of these four methods shown in our study are unknown. We inevitably lost a small number of genes in the calculation of ICS. Nevertheless, the significance of the results remains very promising. Moreover, the usefulness of predictive models is also limited by sequencing platforms, sequencing batches and data normalization methods.

Some studies have investigated the mechanisms by which Nifedipine and Amlodipine act on AAA tissue, controlling for the effects of blood pressure. Nifedipine can inhibit AAA by preserving eNOS coupling activity [78, 79]. It has been demonstrated that *AHR* deficiency attenuates high-fat diet-induced vascular dysfunction by improving eNOS/NO signaling [80]. Topical infusion of amlodipine reduces aortic dilation, but the underlying mechanism is unknown [81]. In contrast, amlodipine increased MMP-9 activity in the lesioned segments [82]. Additionally, there is a lack of studies that can well assess the superiority or inferiority of antihypertensive drugs. It may be that the benefits of lowering blood pressure on AAA have reduced research interest.

In summary, we identified 38 DEMGs that may be involved in AAA. This gene cluster is involved in regulating the maximum vessel diameter, degree of immunoinflammatory infiltration, and strength of the local vessel wall in AAA. The prognostic model we developed can accurately identify the AAA subtypes that tend to rupture.

## Supporting information

S1 File Contains supporting figures and tables. (DOCX)
