# Supplementary material for: Identification of abdominal aortic aneurysm subtypes based on mechanosensitive genes
Source: PLoS One. 2024 Feb 9;19(2):e0296729. doi: 10.1371/journal.pone.0296729 (PMC10857568; doi:10.1371/journal.pone.0296729)
Supplement: S1 File — (DOCX) [file pone.0296729.s001.docx]

**Section 1**

Table S1. Information for selected GEO microarray data sets.

| GSE accession | Year | Platform | Samples | Country | Demographic data | | | | |
| --- | --- | --- | --- | --- | --- | --- | --- | --- | --- |
|  |  |  |  |  | Source of specimen | Aneurysmal diameter | Sex | Age | Others |
| GSE7084 | 2007 | GPL2507 | AAA: Control=7:8 | USA | open surgery to treat AAA; donor | NA | DA | DA | NA |
| GSE47472 | 2013 | GPL10558 | AAA neck: Control=14:8 | Australia | open surgery to treat AAA; donor | NA | NA | NA | NA |
| GSE57691 | 2015 | GPL10558 | AAA: Control=49:10 | Australia | open surgery to treat AAA; donor | NA | NA | NA | Larger AAA and small AAA |
| GSE205071 | 2022 | GPL10558 | AAA high wall stress: low wall stress=12:12 | Germany | NA | DA | DA | DA | NA |
| GSE165470 | 2021 | GPL23126 | AAA (Regional Aortic Weakness differences) | Canada | open surgery to treat AAA | NA | NA | NA | RAW |
| GSE98278 | 2018 | GPL10558 | Intermediate-size AAA: Large AAA=15:16 | Germany | NA | NA | NA | NA | NA |

AAA:abdominal aortic aneurysm; NA: Not available ; DA: Data is available.

Table S2. MSGs list.

| PIEZO1,PIEZO2,PIEZO1P1,PIEZO1P2,TMEM63A,TRPV4,TMEM63B,TMEM150C,TMC2,TRPA1,TMC4,TMC5,TMC3,TMC7,KCNK4,SCN5A,TMEM120A,SNTG2,TMC1,KCNK2,CFTR,CACNA1C,TRPV1,KCNMA1,PTK2,CD47,TRPM7,KCNK10,VCL,CACNA1B,TRPC6,RUNX2,TMEM150A,TMEM150B,PANX1,PTN,YAP1,THBS1,PKD2,TRPC1,CAMKK2,PXN,TLN1,GTF2I,ASIC2,TRPV2,WWTR1,DNAH8,ITGB1,KNG1,PIK3CG,GRM4,SLC8A1,P2RX3,HOTAIR,CTNNB1,EGF,F2R,ASIC3,FOXP2,MIR140,STAT3,ADAM17,NOTCH3,PLA2G4A,AHR,CAPN2,CDK1,EGR1,GABBR1,JUN,PLA2G2A,CYP1A2,ATF3,CANT1,LPP,ANO1,MRTFA,KLF2,RHOD,TMC6,TMC8,ITGB3,HSPB1,CASP3,FN1,KCNQ1,CAV1,DES,ITGAV,COMP,P2RY2,CRHR1,PLCB2,CACNA1I,FLNC,GJA8,BCAR1,TIMP3,GJA3,MYF5,MYOG,IAPP,PLPP3,TAFAZZIN,UCN,MIR181B1,MIR181B2,MIR103A1,JAK2,JAG1,ITGA5,ROCK1,CD55,GATA2,TRPV6,GADD45A,SELE,XPC,CCR7,ADGRE5,ANXA6,KIF2C,ANKRD1,CCK,KDM3A,MYPN,NTS,SLC24A3,ACKR4,MIR126,PKD1,RHOA,PTGS2,PECAM1,GJA1,KCNN4,LMNA,GP1BA,P2RX7,ZYX,TES,TNF,EDN1,FLNA,PTGS1,CD2AP,MIP,KRIT1,HOXA5,SUN2,USH2A,KLF3,ASIC5,EZH2,MMP2,TGFB1,GRIN1,HGF,ITGA2B,MAPK1,MAPK14,SRC,TNFSF11,ICAM1,IL6,MMP14,VIM,ACTN1,ITGA6,BMP4,ENPP1,GNAQ,MYH9,NOS2,SCNN1A,VWF,YES1,AQP1,GNA11,TTN,CDH5,SPP1,CALCA,DMD,MYH10,VCAM1,RARS1,SIRPA,TWIST1,PCSK2,TAGLN,VASP,COX5A,IGFBP5,KCNQ4,SRF,ATP11A,LIMS2,NUP210,PCDH15,SLC44A2,FSTL3,P2RY13,APBB1IP,SLC17A9,UBR4,MAP7,NEAT1,ERK1,ERK2,TFPI2,KLF2,NRF2,HIF1A,NFKB1 |
| --- |

Table S3. Software used in the study.

| Software | Version | Use | Source |
| --- | --- | --- | --- |
| Python | 3.8.8 | Platform | https://www.python.org/ |
| R | 4.2.1 | Platform and statistical  analysis | https://www.r-project.org/ |
| sva | 3.44.0 | Integration of data sets | https://www.bioconductor.org/packages/release/bioc/html/sva.html |
| limma | 3.52.3 | Identification of genes | https://www.bioconductor.org/packages/release/bioc/html/limma.html |
| STRING | 11.5 | PPI analysis | https://string-db.org/ |
| Cytoscape | 3.9.1 | Platform | https://cytoscape.org/ |
| SPSS | 23.0.0.0 | Platform | https://www.ibm.com/spss |
| estimate | 1.0.13 | Immune environment analysis | https://bioinformatics.mdanderson.org/estimate/rpackage.html |
| WGCNA | 1.71 | Identification of genes | https://horvath.genetics.ucla.edu/html/CoexpressionNetwork/Rpackages/WGCNA/ |
| corrplot | 0.92 | Relevance analysis | https://cran.r-project.org/web/packages/corrplot/index.html |
| Sangerbox | 3.0 | Platform | http://sangerbox.com/home.html |
|  |  |  |  |

Table S4. 38 DEMGs.

|  | Genes |
| --- | --- |
| Downregulated | KCNMA1, LPP, HSPB1, PKD1, GJA1, LIMS2, PKD2, PLA2G2A, GNA11, FLNC, JAG1, DES, JAK2, MYH10, HOXA5, TRPC1, IGFBP5, ROCK1, TAGLN, GADD45A, CAV1, AQP1, VCL, FSTL3, YAP1, SLC24A3, ACTN1, NOTCH3, TWIST1, FLNA |
| Upregulated | IL6, CCR7, PTGS2, SPP1, NUP210, PLCB2, KCNN4, AHR |

Table S5. hub genes under four algorithms.

| Algorithms | Genes (Sorted by score) |
| --- | --- |
| Degree | VCL, IL6, CAV1, TAGLN, GJA1, FLNA, PTGS2, SPP1, JAK2, JAG1 |
| MNC | VCL, IL6, CAV1, TAGLN, PTGS2, GJA1, FLNA, JAG1, SPP1, ACTN1 |
| Closeness | VCL, CAV1, IL6, GJA1, TAGLN, SPP1, JAK2, FLNA, PTGS2, PKD1 |
| MCC | VCL, TAGLN, IL6, CAV1, FLNA, ACTN1, PTGS2, MYH10, GJA1, SPP1 |
| Intersecting genes | FLNA, CAV1, TAGLN, SPP1, VCL, GJA1, IL6, PTGS2 |

|  | |
| --- | --- |
|  |  |
| Table S6. Immune cell scores for each AAA sample (merged dataset).   \| **Sample** \| **ImmuneScore** \| **Cluster** \| \| --- \| --- \| --- \| \| GSE57691_GSM1386783 \| 1494.488958 \| 1 \| \| GSE57691_GSM1386785 \| 2124.062546 \| 1 \| \| GSE57691_GSM1386788 \| 2475.992679 \| 1 \| \| GSE57691_GSM1386789 \| 1963.079122 \| 1 \| \| GSE57691_GSM1386792 \| 2867.277479 \| 1 \| \| GSE57691_GSM1386793 \| 3306.829991 \| 1 \| \| GSE57691_GSM1386795 \| 2415.311081 \| 1 \| \| GSE57691_GSM1386796 \| 3014.906903 \| 1 \| \| GSE57691_GSM1386797 \| 2465.874667 \| 1 \| \| GSE57691_GSM1386800 \| 2938.859333 \| 1 \| \| GSE57691_GSM1386803 \| 2279.45391 \| 1 \| \| GSE57691_GSM1386805 \| 1411.61172 \| 1 \| \| GSE57691_GSM1386807 \| 2723.486308 \| 1 \| \| GSE57691_GSM1386808 \| 2988.75791 \| 1 \| \| GSE57691_GSM1386812 \| 829.2541573 \| 1 \| \| GSE57691_GSM1386813 \| 1478.934421 \| 1 \| \| GSE57691_GSM1386816 \| 2689.149926 \| 1 \| \| GSE57691_GSM1386818 \| 2391.3778 \| 1 \| \| GSE57691_GSM1386820 \| 1065.484555 \| 1 \| \| GSE57691_GSM1386822 \| 2019.29462 \| 1 \| \| GSE57691_GSM1386825 \| 2370.848142 \| 1 \| \| GSE57691_GSM1386829 \| 3138.779621 \| 1 \| \| GSE57691_GSM1386831 \| 881.9014625 \| 1 \| \| GSE7084_GSM170550 \| 2873.385683 \| 1 \| \| GSE7084_GSM170554 \| 2884.840088 \| 1 \| \| GSE7084_GSM170555 \| 2769.707975 \| 1 \| \| GSE7084_GSM170563 \| 3050.757542 \| 1 \| \| GSE57691_GSM1386784 \| 2606.16843 \| 2 \| \| GSE57691_GSM1386786 \| 2776.06199 \| 2 \| \| GSE57691_GSM1386787 \| 2207.395606 \| 2 \| \| GSE57691_GSM1386790 \| -247.2965661 \| 2 \| \| GSE57691_GSM1386791 \| 1301.970663 \| 2 \| \| GSE57691_GSM1386794 \| 3196.3749 \| 2 \| \| GSE57691_GSM1386798 \| -194.4103235 \| 2 \| \| GSE57691_GSM1386799 \| -955.6232973 \| 2 \| \| GSE57691_GSM1386801 \| -850.9449587 \| 2 \| \| GSE57691_GSM1386802 \| 2336.311688 \| 2 \| \| GSE57691_GSM1386804 \| 2511.259641 \| 2 \| \| GSE57691_GSM1386806 \| 2442.04999 \| 2 \| \| GSE57691_GSM1386809 \| 2318.555869 \| 2 \| \| GSE57691_GSM1386810 \| 2751.404438 \| 2 \| \| GSE57691_GSM1386811 \| 2281.76925 \| 2 \| \| GSE57691_GSM1386814 \| 2245.863045 \| 2 \| \| GSE57691_GSM1386815 \| 2374.514303 \| 2 \| \| GSE57691_GSM1386817 \| 512.993373 \| 2 \| \| GSE57691_GSM1386819 \| 1508.362235 \| 2 \| \| GSE57691_GSM1386821 \| 914.5162816 \| 2 \| \| GSE57691_GSM1386823 \| 1952.157135 \| 2 \| \| GSE57691_GSM1386824 \| 2556.224282 \| 2 \| \| GSE57691_GSM1386826 \| 2207.710054 \| 2 \| \| GSE57691_GSM1386827 \| 1407.460178 \| 2 \| \| GSE57691_GSM1386828 \| 1800.17459 \| 2 \| \| GSE57691_GSM1386830 \| 1195.316868 \| 2 \| \| GSE7084_GSM170551 \| 1909.193238 \| 2 \| \| GSE7084_GSM170552 \| 2442.041735 \| 2 \| \| GSE7084_GSM170553 \| 3002.560906 \| 2 \| | |

Table S7. ICS for each AAA sample (merged dataset).

| Sample | ICS | Cluster | Sample | ICS | Cluster |
| --- | --- | --- | --- | --- | --- |
| GSM1386783 | 0.352065 | 1 | GSM1386786 | 0.312801 | 2 |
| GSM1386785 | 0.407076 | 1 | GSM1386787 | 0.02241 | 2 |
| GSM1386788 | 0.477279 | 1 | GSM1386790 | -0.5123 | 2 |
| GSM1386789 | 0.664566 | 1 | GSM1386791 | -0.15638 | 2 |
| GSM1386792 | 0.970311 | 1 | GSM1386794 | 0.942105 | 2 |
| GSM1386793 | 0.877447 | 1 | GSM1386798 | -0.18093 | 2 |
| GSM1386795 | 0.676282 | 1 | GSM1386799 | -0.35791 | 2 |
| GSM1386796 | 0.801863 | 1 | GSM1386801 | -0.31045 | 2 |
| GSM1386797 | 1.05526 | 1 | GSM1386802 | 0.262642 | 2 |
| GSM1386800 | 0.782005 | 1 | GSM1386804 | 0.171658 | 2 |
| GSM1386803 | 0.32852 | 1 | GSM1386806 | 0.178986 | 2 |
| GSM1386805 | 0.374563 | 1 | GSM1386809 | 0.341716 | 2 |
| GSM1386807 | 0.394849 | 1 | GSM1386810 | 0.289987 | 2 |
| GSM1386808 | 0.64582 | 1 | GSM1386811 | 0.308667 | 2 |
| GSM1386812 | 0.152468 | 1 | GSM1386814 | 0.181795 | 2 |
| GSM1386813 | 0.336157 | 1 | GSM1386815 | -0.01187 | 2 |
| GSM1386816 | 0.555436 | 1 | GSM1386817 | 0.017104 | 2 |
| GSM1386818 | 0.564077 | 1 | GSM1386819 | 0.116726 | 2 |
| GSM1386820 | 0.191071 | 1 | GSM1386821 | -0.08649 | 2 |
| GSM1386822 | 0.482864 | 1 | GSM1386823 | 0.647514 | 2 |
| GSM1386825 | 0.523152 | 1 | GSM1386824 | 0.738424 | 2 |
| GSM1386829 | 0.780173 | 1 | GSM1386826 | 0.309153 | 2 |
| GSM1386831 | 0.20895 | 1 | GSM1386827 | 0.110411 | 2 |
| GSM170550 | 0.552758 | 1 | GSM1386828 | 0.501322 | 2 |
| GSM170554 | 1.541408 | 1 | GSM1386830 | 0.00613 | 2 |
| GSM170555 | 1.437146 | 1 | GSM170551 | 0.169869 | 2 |
| GSM170563 | 0.984482 | 1 | GSM170552 | 0.697525 | 2 |
| GSM1386784 | 0.456626 | 2 | GSM170553 | 0.569682 | 2 |

Table S8. Workflow for the development of an Atherosclerosis score.

|  | Description | Important parameter settings |
| --- | --- | --- |
| Step 1 | Expression matrix acquisition and organization | 9 samples of arterial occlusive disease, and 10 control samples |
| Step 2 | Differential gene screening | adjusted p-value ＜ 0.05 and \|log 2 (FC)\| > 1.5 |
| Step 3 | PPI network construction | confidence score of 0.9 |
| Step 4 | Identification of intersecting genes under four algorithms | degree, MNC, closeness, and MCC algorithms |
| Step 5 | Model construction using ‘linear’ SVM models | Area under the curve for assessing model performance |
| Atherosclerosis score = (-0.45438071 ×FAU) + (0.55211903 ×RPS13) + (-0.17563046 ×RPS15A) + (0.47586899 ×RPS18) +( -1.41973587 ×RPS24) + (-1.62142618 ×RPS25) + (-1.37834413 ×RPS6) | | |

Table S9. Intersecting genes of the sDEGs, the highest correlated modular genes, and the MSGs (Bolded genes are non-DEMG).

| LPP TAGLN **EDN1** HSPB1 GJA1 LIMS2 GADD45A CAV1 FLNC DES MYH10 YAP1 SLC24A3 ACTN1 IGFBP5 |
| --- |

Table S10. Mechanical sensitivity scores for each AAA sample (merged dataset).

| Sample | Score | Cluster | Sample | Score | Cluster |
| --- | --- | --- | --- | --- | --- |
| GSM1386783 | -2.96419 | 1 | GSM1386784 | -9.44359 | 2 |
| GSM1386785 | -1.32781 | 1 | GSM1386786 | -9.35649 | 2 |
| GSM1386788 | -3.99406 | 1 | GSM1386787 | -10.7527 | 2 |
| GSM1386789 | -2.6468 | 1 | GSM1386790 | -10.3079 | 2 |
| GSM1386792 | -4.0646 | 1 | GSM1386791 | -13.4267 | 2 |
| GSM1386793 | -0.57613 | 1 | GSM1386794 | -5.9942 | 2 |
| GSM1386795 | -1.19326 | 1 | GSM1386798 | -8.92561 | 2 |
| GSM1386796 | 0.498757 | 1 | GSM1386799 | -9.3248 | 2 |
| GSM1386797 | -1.74997 | 1 | GSM1386801 | -10.5343 | 2 |
| GSM1386800 | -2.08305 | 1 | GSM1386802 | -5.99413 | 2 |
| GSM1386803 | -3.99441 | 1 | GSM1386804 | -10.763 | 2 |
| GSM1386805 | -2.40121 | 1 | GSM1386806 | -10.4913 | 2 |
| GSM1386807 | -3.54595 | 1 | GSM1386809 | -9.9656 | 2 |
| GSM1386808 | -3.43503 | 1 | GSM1386810 | -11.3075 | 2 |
| GSM1386812 | -2.62233 | 1 | GSM1386811 | -8.82149 | 2 |
| GSM1386813 | -3.81699 | 1 | GSM1386814 | -10.1631 | 2 |
| GSM1386816 | -3.99441 | 1 | GSM1386815 | -12.6725 | 2 |
| GSM1386818 | 0.493099 | 1 | GSM1386817 | -5.64266 | 2 |
| GSM1386820 | -3.27278 | 1 | GSM1386819 | -12.1883 | 2 |
| GSM1386822 | -2.782 | 1 | GSM1386821 | -12.1528 | 2 |
| GSM1386825 | 0.985735 | 1 | GSM1386823 | -8.07138 | 2 |
| GSM1386829 | -3.99409 | 1 | GSM1386824 | -8.36117 | 2 |
| GSM1386831 | -2.60799 | 1 | GSM1386826 | -7.21939 | 2 |
| GSM170550 | -2.31796 | 1 | GSM1386827 | -5.99441 | 2 |
| GSM170554 | 1.584555 | 1 | GSM1386828 | -5.9507 | 2 |
| GSM170555 | -0.12693 | 1 | GSM1386830 | -6.04704 | 2 |
| GSM170563 | -3.9948 | 1 | GSM170551 | -5.9945 | 2 |
| GSM1386783 | -2.96419 | 1 | GSM170552 | -6.29718 | 2 |

**Section 2**


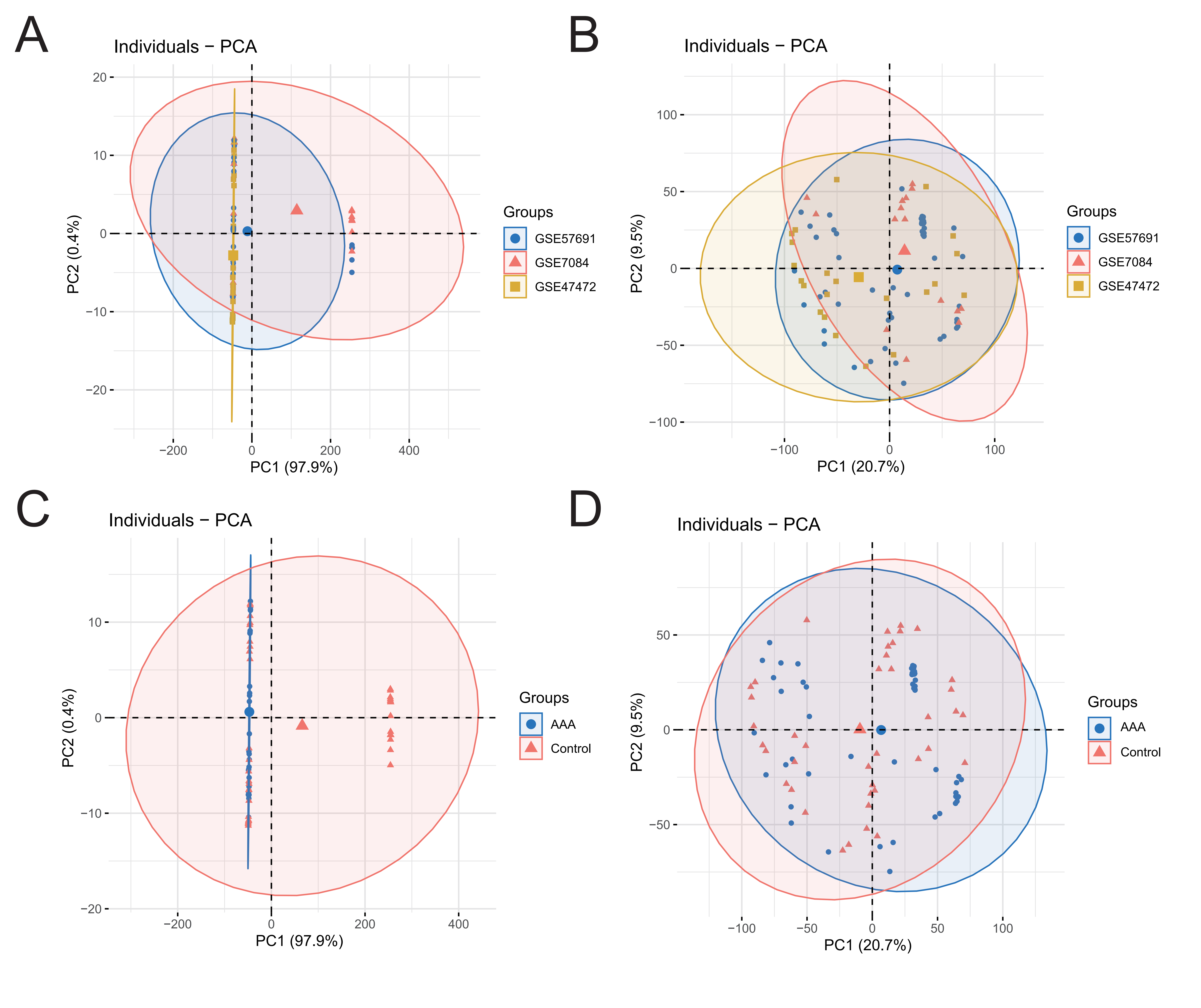


Figure S1. (A) and (B) Data distribution of the datasets (GSE57691, GSE7084, GSE47472) before and after integration. (C) and (D) Data distribution of AAA and control samples before and after integration.


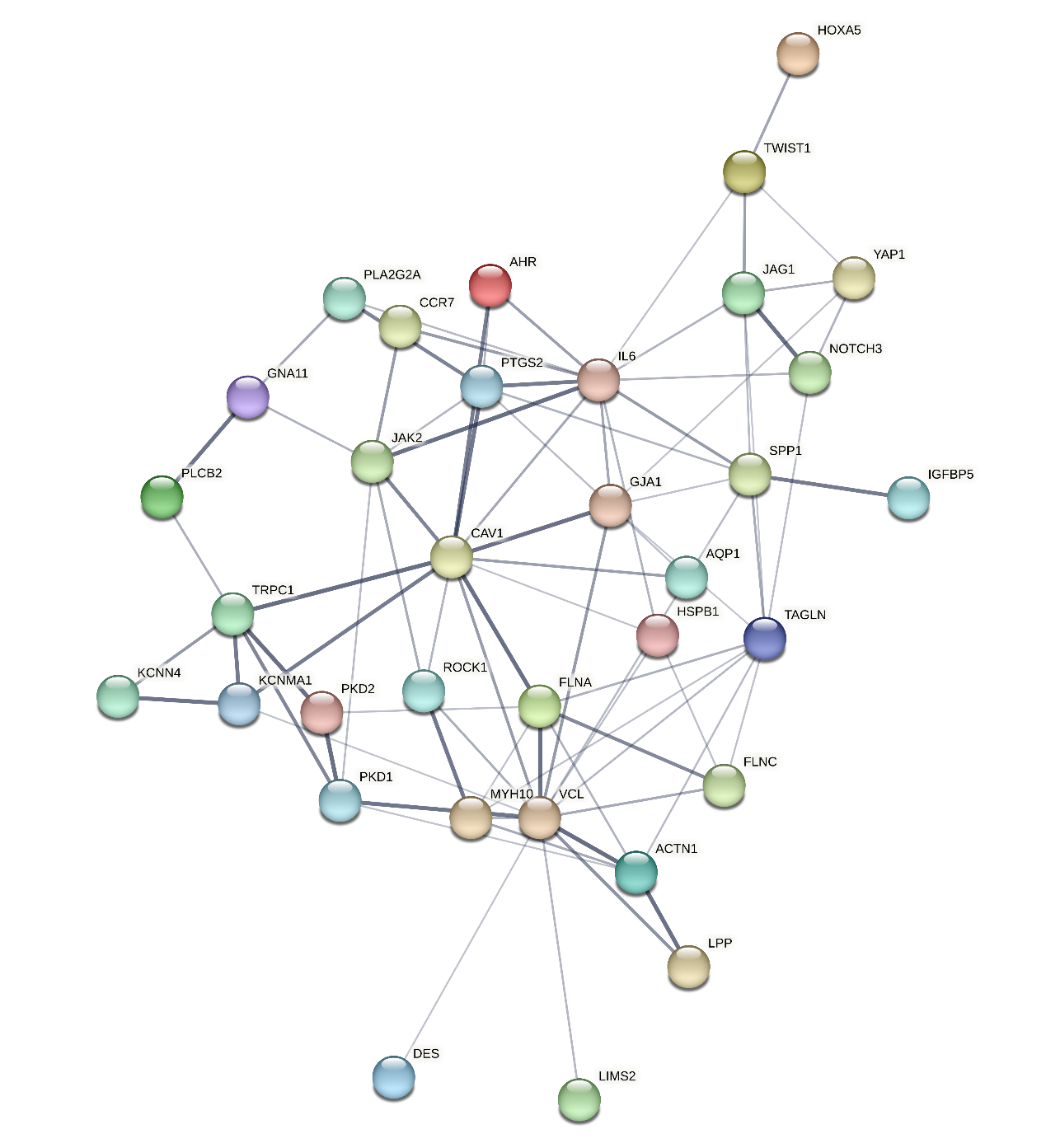


Figure S2. PPI network for DEMGs. Line thickness indicates the strength of data support. The colors represent different proteins.


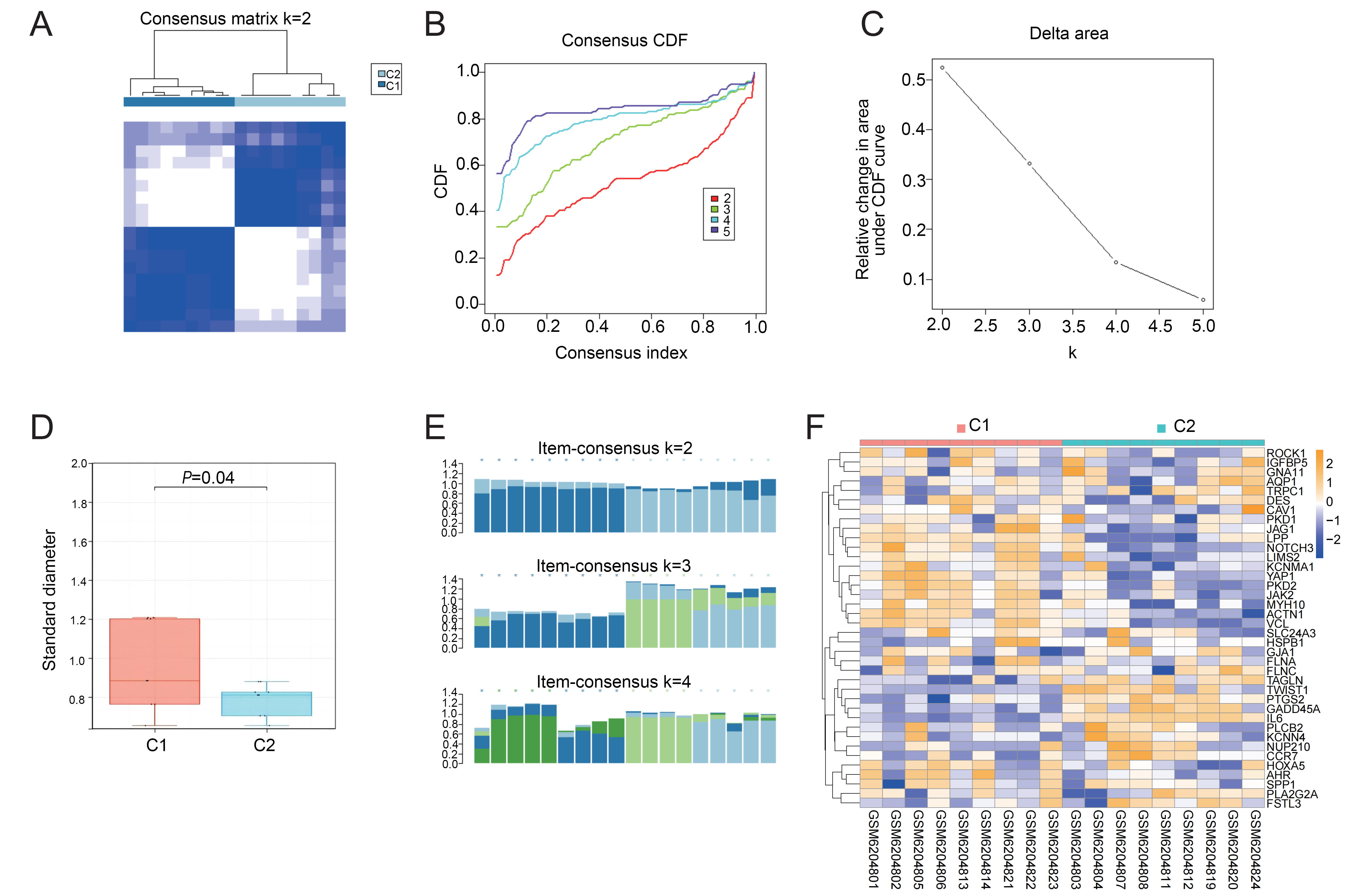


Figure S3. Consensus clustering analysis of the GSE205071. (A) Heatmap of the co-occurrence ratio matrix of AAA samples. (B) Consensus clustering CDF for k=2-5. (C) Relative alterations in the area under the cumulative distribution function (CDF) curve for k = 2-5. (D) Comparison of Standard diameter between subgroups. (E) Item-Consensus plot for k = 2, 3, 4. The longitudinal coordinate is the mean of pairwise consensus value. (F) Expression level heatmap and hierarchical clustering of DEMGs in the AAA subgroups.


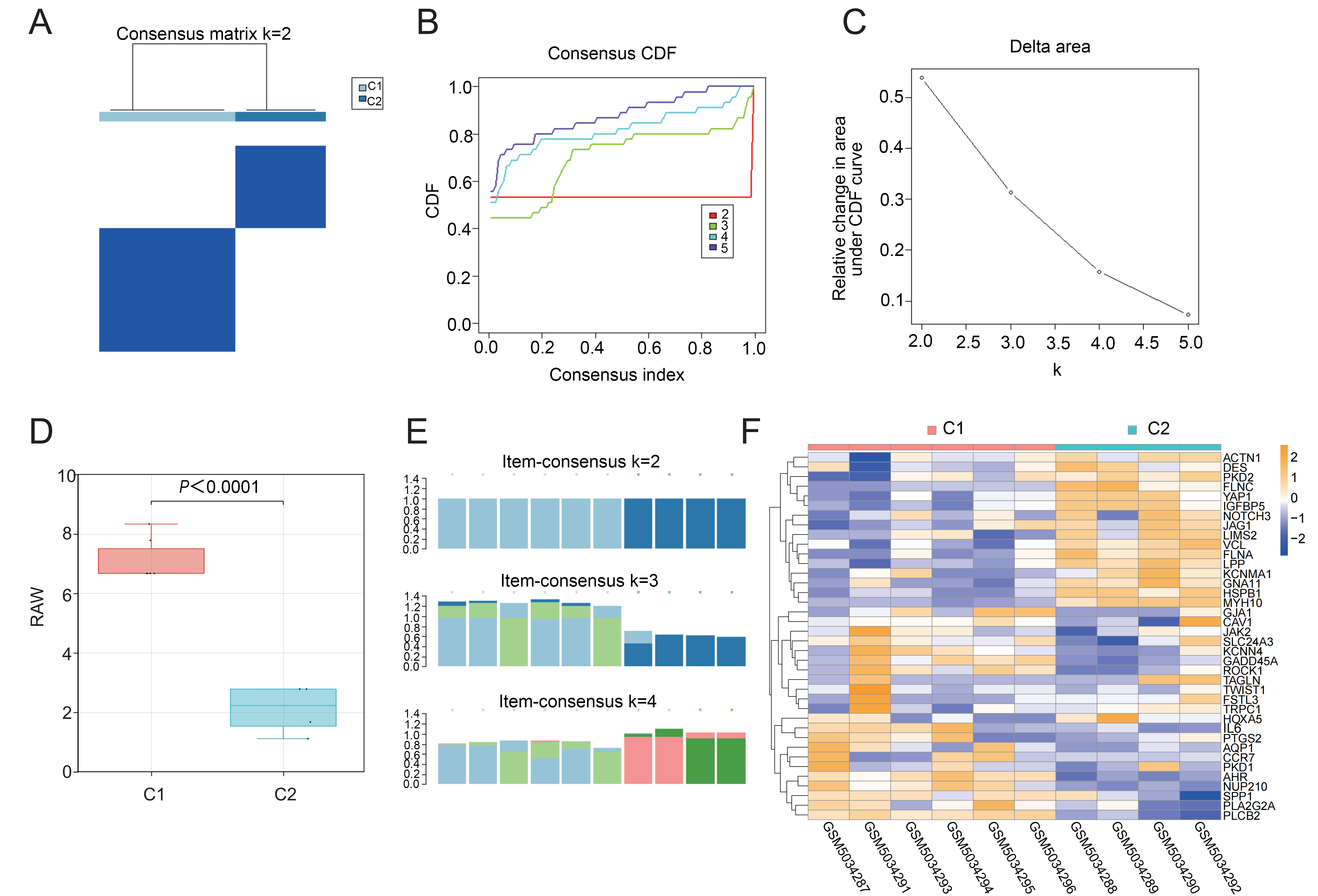


Figure S4. Consensus clustering analysis of the GSE165470. (A) Heatmap of the co-occurrence ratio matrix of AAA samples. (B) Consensus clustering CDF for k=2-5. For k = 2, the CDF curve for consensus index between 0.1 and 0.9 drops very little, indicating that the classification is very reliable. (C) Relative alterations in the area under the cumulative distribution function (CDF) curve for k = 2-5. (D) Comparison of RAW between subgroups. (E) Item-Consensus plot for k = 2, 3, 4. The longitudinal coordinate is the mean of pairwise consensus value. (F) Expression level heatmap and hierarchical clustering of DEMGs in the AAA subgroups.


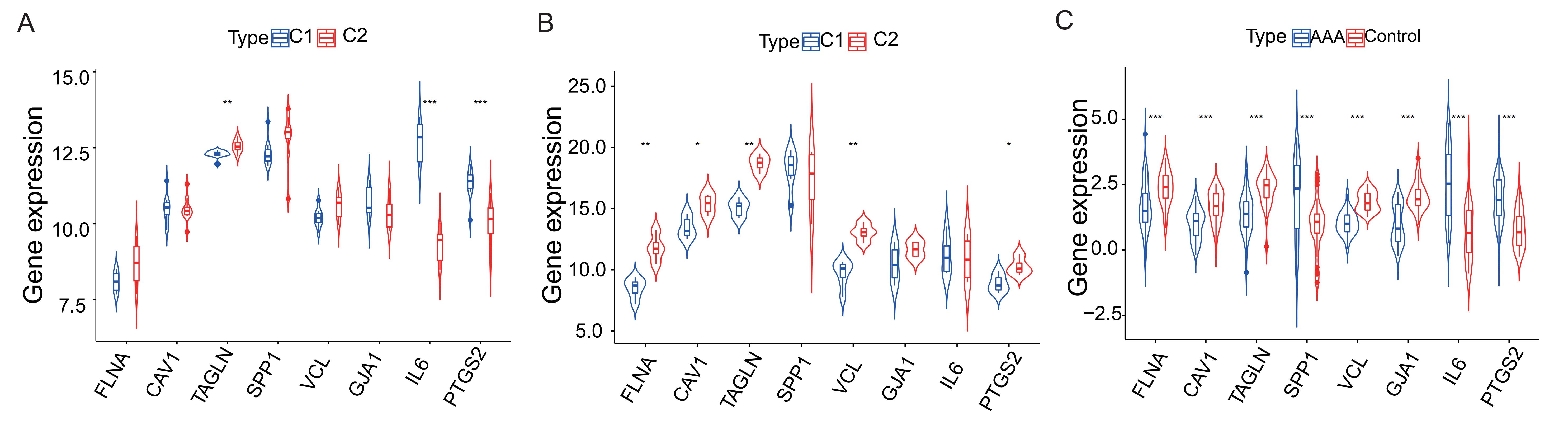


Figure S5. (A) Intergroup comparison of hub gene expression in GSE205071. (B) Intergroup comparison of hub gene expression in GSE165470. (C) Intergroup comparison of hub gene expression in merged dataset. *^🟉^* *P* < 0.05, *^🟉🟉^* *P* < 0.01, *^🟉🟉🟉^* *P* < 0.001, Blank: no significance.


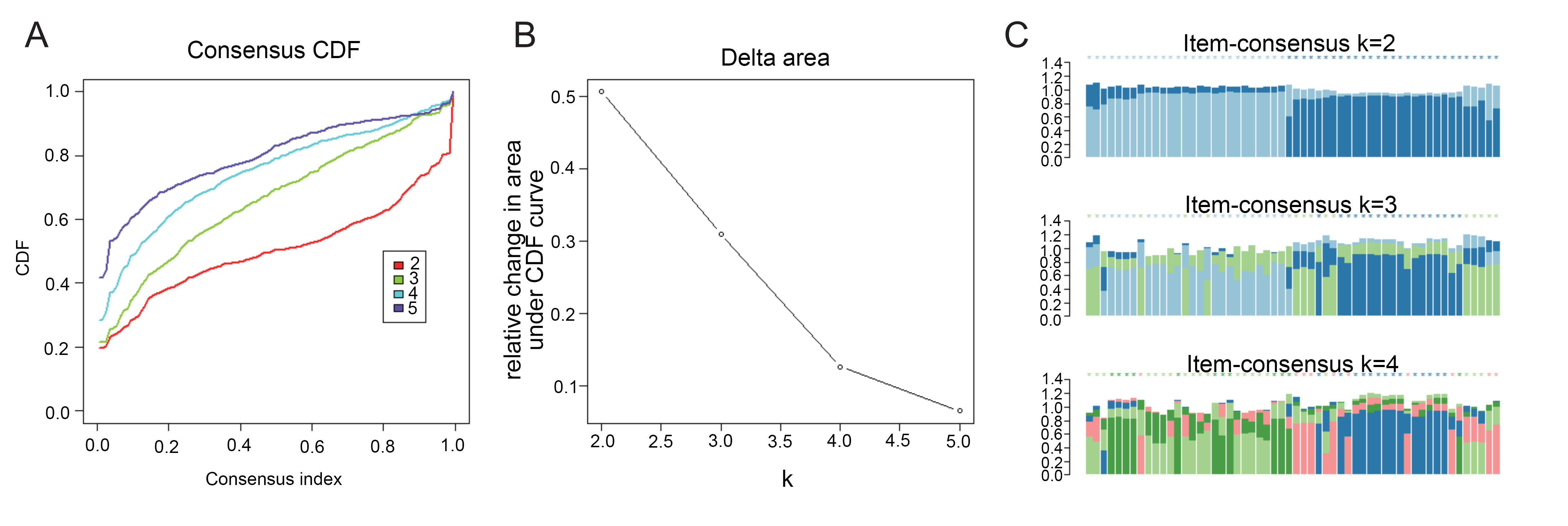


Figure S6. Consensus clustering analysis of AAA case in merged dataset. (A) Consensus clustering CDF for k=2-5. (B) Relative alterations in the area under the cumulative distribution function (CDF) curve for k = 2-5. (C) Item-Consensus plot for k = 2, 3, 4. The longitudinal coordinate is the mean of pairwise consensus value.





Figure S7. Construction of an atherosclerosis score based on GSE57691. (A) Expression level heatmap of DEGs in the non-aneurysm control and atherosclerosis groups. AOD, arterial occlusive disease. (B) ROC curves for tri-fold cross-validation show the performance of the SVM model.
